# Targeted Temperature Management in Cardiogenic Shock Survivors of Cardiac Arrest: A Systematic Review and Meta-Analysis

**DOI:** 10.7759/cureus.102336

**Published:** 2026-01-26

**Authors:** Hossam A Kordi, Khaled A Soliman, Farrukh Ameer, Ahmed Osman Hassan Ali, Ahmad M AaL Ibrahim, Bandar S Alshreef, Dalya Alabdurab Alnabi, Moathe Alolayan, Gehad K Mousa, Ragy Ragab, Rodyna Mostafa, Reem F Al.Maghthawi, Jood Alsaadi, Faisal I Alfadda, Osama F Almabadi

**Affiliations:** 1 Emergency Medicine, Abha International Private Hospital, Abha, SAU; 2 Emergency Medicine, Armed Forces Hospital Southern Region, Khamis Mushait, SAU; 3 Cardiology, Dr. Soliman Fakeeh Hospital, Riyadh, SAU; 4 Critical Care, Dr. Soliman Fakeeh Hospital, Riyadh, SAU; 5 Emergency Medicine, Dr. Soliman Fakeeh Hospital, Riyadh, SAU; 6 Emergency Medicine, Riyadh Hospital, Riyadh, SAU; 7 Healthcare Management, College of Applied Medical Sciences, Shaqra University, Shaqra, SAU; 8 Medicine, Qatif Central Hospital, Al Qatif, SAU; 9 Medicine, College of Medicine, King Saud University, Riyadh, SAU; 10 Intensive Care, National Heart Institute, Giza, EGY; 11 Intensive Care, Zagazig University, Zagazig, EGY; 12 Cardiology, City University of New York (CUNY) School of Medicine, New York City, USA; 13 Medicine, College of Medicine and Surgery, Taibah University, Madinah, SAU; 14 Medicine, Ibn Sina National College, Jeddah, SAU; 15 Medicine, College of Medicine, Qassim University, Buraydah, SAU; 16 Medicine and Surgery, University of Jeddah, Jeddah, SAU

**Keywords:** cardiac arrest, cardiogenic shock, hypothermia, meta-analysis, mortality, targeted temperature management

## Abstract

Targeted temperature management (TTM) is the established standard of care for comatose patients following cardiac arrest, but its safety profile and effectiveness in the subset of patients developing cardiogenic shock are not well defined. Benefits of neuroprotection may be offset by hypothermia-induced hemodynamic instability. This review aimed to evaluate the impact of TTM on mortality and neurological outcomes, specifically in cardiac arrest survivors with cardiogenic shock. PubMed, EMBASE, and Cochrane Central were searched from inception to December 2025 for randomized controlled trials (RCTs) and observational studies comparing TTM (32°C-34°C) versus normothermia or no temperature control in adults with post-cardiac arrest cardiogenic shock. The primary outcome was all-cause mortality. Secondary outcomes included favorable neurological function and lactate clearance. Data were pooled using a random-effects model (DerSimonian-Laird). Certainty of evidence was assessed using GRADE (Grading of Recommendations Assessment, Development, and Evaluation). Five studies involving 1,446 patients were included (two RCT sub-analyses and three observational studies). The pooled risk ratio (RR) for all-cause mortality was 1.02 (95% confidence interval (CI) 0.89-1.17; p = 0.76), indicating no significant survival benefit with TTM. Significant heterogeneity was observed (I^2^ = 47.9%), driven by a divergence between observational studies, which favored TTM (RR 0.90; 95% CI 0.63-1.27), and RCTs, which showed a trend toward harm or neutrality (RR 1.05; 95% CI 0.86-1.28). TTM was not associated with improved neurological outcomes or lactate clearance. Meta-regression revealed a temporal trend where more recent, higher-quality studies reported less favorable outcomes for TTM. In survivors of cardiac arrest complicated by cardiogenic shock, TTM at 32°C-34°C was not associated with reduced mortality compared to normothermia. The apparent benefit seen in early observational data is not supported by recent randomized evidence, which raises concerns regarding hemodynamic tolerability. Strict normothermia may be a safer therapeutic strategy in this high-risk population. The certainty of the evidence is very low.

## Introduction and background

Globally, out-of-hospital cardiac arrest (OHCA) remains a primary cause of death and significant neurological impairment. While achieving return of spontaneous circulation (ROSC) is the immediate objective of resuscitation efforts, the subsequent post-cardiac arrest syndrome is characterized by a complex combination of systemic ischemia-reperfusion injury, brain injury, and myocardial dysfunction [1]. This post-cardiac arrest syndrome is frequently complicated by cardiogenic shock (CS), a state of critical end-organ hypoperfusion caused by primary pump failure. Targeted temperature management (TTM) has served as the cornerstone of neuroprotective care for comatose survivors of cardiac arrest, based on landmark trials demonstrating improved survival and neurological outcomes with mild hypothermia (32°C-34°C) [2,3].

However, the therapeutic landscape has evolved, as more recent large-scale randomized controlled trials (RCTs), such as the TTM trial [4] and TTM-2 trial [5], have suggested that targeting normothermia (36°C) or fever prevention (<37°C) results in survival and neurological outcomes comparable to induced hypothermia (33°C). The HYPERION trial demonstrated the functional benefit of moderate hypothermia (33°C), specifically in patients with non-shockable rhythms [6], suggesting that specific phenotypes of patients with cardiac arrest may respond differently to temperature interventions.

Among these phenotypes, patients presenting with CS following cardiac arrest represent a particularly high-risk subgroup [7]. The post-resuscitation period in these patients is complicated by myocardial stunning and hemodynamic instability, often requiring vasopressor support or mechanical circulatory assistive devices [8,9]. The application of therapeutic hypothermia in this cohort presents a distinct physiological paradox that requires further investigation. While hypothermia aims to mitigate cerebral injury, it creates a distinct physiological paradox in CS. Therapeutic cooling is known to induce bradycardia and increase systemic vascular resistance (SVR) (afterload); in a heart with stunned myocardium, these changes may compromise cardiac output and exacerbate end-organ hypoperfusion [10]. The pivotal early trials establishing TTM specifically excluded patients with profound CS [2,3], leaving a critical knowledge gap regarding the safety and efficacy of hypothermia in this population. Furthermore, while these landmark trials focused on OHCA, the hemodynamic risks associated with cooling, specifically the suppression of cardiac contractility, are anticipated to be universally relevant to CS survivors, regardless of whether the arrest etiology was out-of-hospital or in-hospital.

Recent data have challenged the safety of cooling in patients with primary CS, evidenced by the SHOCK-COOL trial, which reported that mild hypothermia provided no hemodynamic advantages in patients suffering from acute myocardial infarction complicated by CS, in the absence of cardiac arrest [10], while post hoc analyses of the TTM-2 [11] and HYPERION [12] trials examined patients with circulatory shock and post-resuscitation hemodynamic instability, yielding conflicting signals regarding the interaction between temperature targets and survival in the presence of shock. Given the physiological complexity and exclusion of patients with severe shock from foundational trials, the optimal temperature target for this specific subpopulation remains undefined. Therefore, this systematic review and meta-analysis of RCTs and high-quality observational studies was conducted to evaluate the efficacy and safety of TTM (32°C-34°C) compared to normothermia or no temperature control in cardiac arrest survivors complicated by CS.

## Review

Methods

Protocol and Registration

This systematic review and meta-analysis was conducted in accordance with the Preferred Reporting Items for Systematic Reviews and Meta-Analyses (PRISMA) guidelines [13]. The study protocol was prospectively registered with the PROSPERO database (CRD420251158341).

Search Strategy and Eligibility Criteria

Major electronic databases were queried, including EMBASE, PubMed/MEDLINE, and the Cochrane Central Register of Controlled Trials (CENTRAL), covering records from their inception through December 2025. The search was restricted to full-text articles published in the English language; conference abstracts and unpublished data were excluded to ensure peer-reviewed data quality. RCTs and high-quality observational studies that met the following PICO criteria were included: (1) Population: adult survivors of cardiac arrest (out-of-hospital or in-hospital) presenting with clinical evidence of CS or requiring vasopressor support post-resuscitation; (2) Intervention: TTM with a hypothermic target (32°C-34°C); (3) Comparator: normothermia, fever prevention (≥36°C), or no temperature control; and (4) Outcomes: primary outcome of all-cause mortality. Secondary outcomes included favorable neurological function (defined as a Cerebral Performance Category (CPC) of 1-2 or modified Rankin Scale (mRS) of 0-3) and adverse hemodynamic events. Lactate clearance was selected as a secondary outcome as it serves as a broadly available biochemical surrogate for end-organ perfusion and the reversal of the shock state. Included studies were screened for overlapping patient populations to prevent double-counting; the RCTs (TTM-2 and HYPERION) and observational registries were confirmed to be distinct cohorts. In addition to electronic searches, the reference lists of all included studies and relevant reviews were manually screened to identify additional eligible articles. Search terms included combinations of Medical Subject Headings (MeSH) and keywords such as “targeted temperature management”, “therapeutic hypothermia”, “cardiogenic shock”, “heart arrest”, and “vasopressor support”.

Risk of Bias Assessment

Two investigators independently assessed the methodological quality of the included studies. For RCTs, the risk of bias was evaluated using the Cochrane Risk of Bias 2 (RoB 2) tool [14]. For non-randomized and observational studies, the risk of bias in non-randomized studies of interventions (ROBINS-I) tool was employed to assess confounding and selection bias [15]. Discrepancies were resolved through consensus or consultation with a third investigator.

Statistical Analysis and Data Synthesis

Data were synthesized using a random-effects meta-analysis model. To address expected variance between studies and clinical heterogeneity regarding shock severity, the DerSimonian-Laird method was employed [16]. To ensure robustness and control type I error rates given the potential for a small number of included studies and unequal sample sizes, the Hartung-Knapp-Sidik-Jonkman (HKSJ) adjustment [17] was applied to calculate confidence intervals (CIs). Raw event data were extracted to calculate unadjusted risk ratios (RRs) to ensure methodological consistency across study designs. Missing data were handled using a complete-case analysis.

Dichotomous outcomes (mortality, favorable neurological status) were expressed as RRs or odds ratios (ORs) with 95% CIs. Mortality outcomes reported at hospital discharge, 30 days, and six months were pooled for the primary analysis. This approach assumes that, consistent with post-cardiac arrest epidemiology, the majority of mortality occurs early due to withdrawal of life support, making these time points clinically comparable. Continuous outcomes were analyzed using weighted mean differences (MDs). Statistical heterogeneity was quantified using the I^2^ statistic and the chi-squared (χ^2^) test for dispersion, where an I^2^ > 50% indicated substantial heterogeneity [18].

Assessment of Bias and Small-Study Effects

Reporting and dissemination biases were evaluated to identify potential selective outcomes. Visual inspection of funnel plots was used to assess small-study effects and publication bias. These visual assessments were statistically tested using Egger’s linear regression and Begg’s rank correlation tests, with a p-value < 0.1 considered indicative of significant asymmetry [19,20] and confirmed using Harbord’s modified test and Peters’ test, which are preferred for binary meta-analyses as they reduce the risk of false-positive indications of bias compared to standard linear regression methods.

Selective outcome reporting was assessed by verifying that outcomes defined in the methods sections of primary studies were fully reported in the results. Given the small number of included studies (n < 10), funnel plot assessments were considered exploratory. Contour-enhanced plots were utilized to differentiate asymmetry caused by publication bias from that caused by heterogeneity.

Robustness and Sensitivity Analyses

To evaluate the stability of the pooled estimates, sensitivity analyses were conducted by sequentially removing individual studies (leave-one-out analysis) and excluding studies with a high risk of bias. Furthermore, the subgroup analysis stratifying by study design (RCT vs. observational) served as a sensitivity analysis for study quality, distinguishing high-quality randomized evidence from lower-quality observational data. Subgroup analyses and meta-regression (moderators) were planned to explore sources of heterogeneity, specifically stratifying by shockable vs. non-shockable rhythms, severity of shock (vasopressor score), and cooling methods (intravascular vs. surface), provided sufficient data were available (n ≥ 10 studies).

Certainty of Evidence

The overall strength of the body of evidence was appraised using the Grading of Recommendations Assessment, Development, and Evaluation (GRADE) framework. Evidence was categorized as high, moderate, low, or very low quality based on the risk of bias, inconsistency, indirectness, imprecision, and publication bias [21].

Results

Search Results and Study Selection

**Figure 1:**
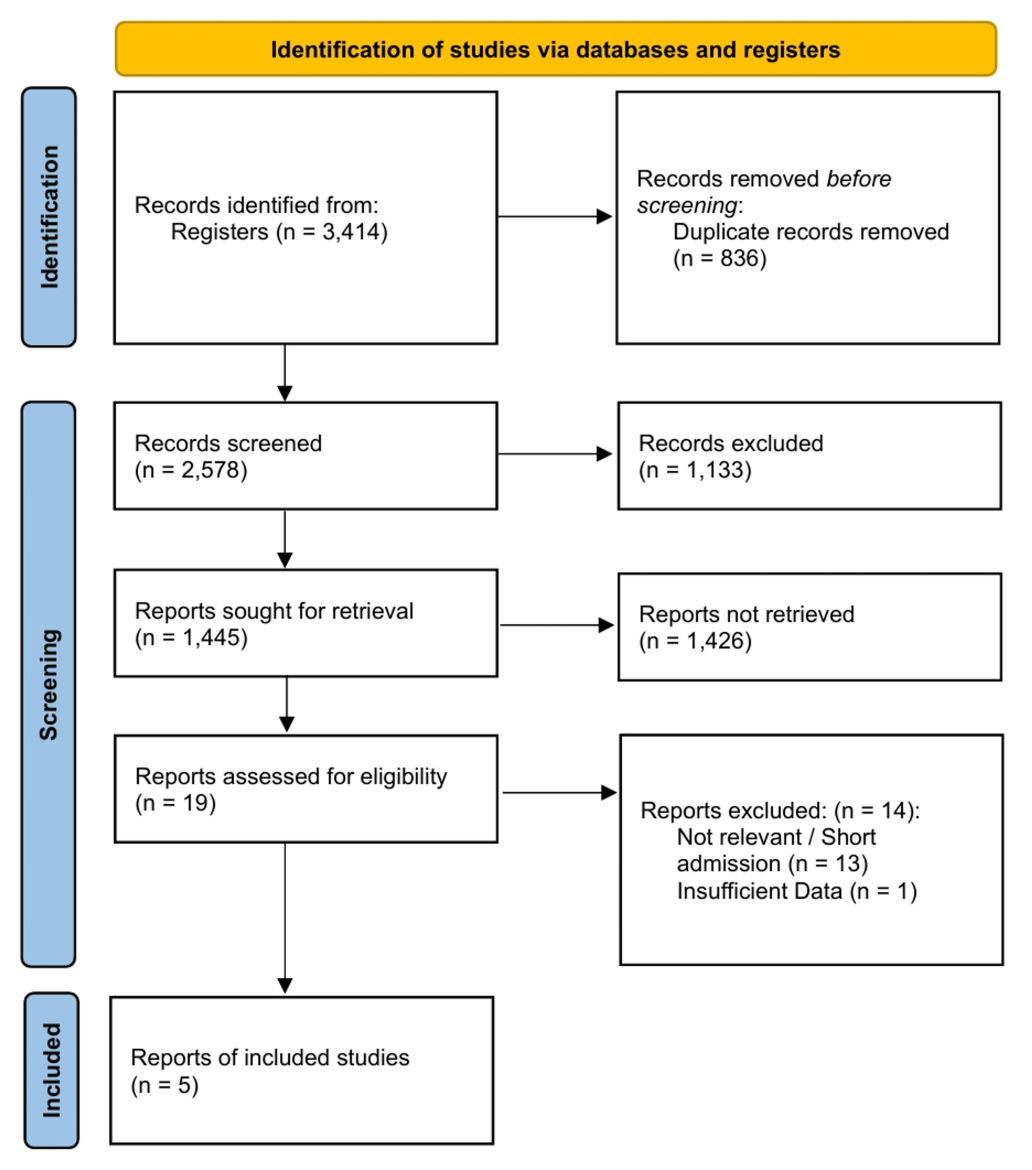
PRISMA flow diagram PRISMA: Preferred Reporting Items for Systematic Reviews and Meta-Analyses

The initial systematic search yielded 3,414 citations in total. After removing 836 duplicates, 2,578 records were screened based on their titles and abstracts. Of these, 2,559 were excluded for being irrelevant to the specific topic of TTM in CS. Nineteen full-text articles were assessed for eligibility. Fourteen studies were excluded for not meeting the inclusion criteria, such as a lack of a specific CS subgroup, wrong comparator, or animal studies. Five studies met the final inclusion criteria for quantitative synthesis [8,9,11,12,22]. The single-arm study by Hovdenes et al. [22] was excluded from the quantitative meta-analysis due to the absence of a control group and was synthesized narratively. The study selection process is detailed in the PRISMA flowchart (Figure 1).

Study Characteristics

The included studies comprised two post hoc analyses of large multicenter RCTs [11,12] and three observational cohort studies [8,9,22]. The total pooled population included 1,446 patients with cardiac arrest complicated by CS or requiring significant vasopressor support. The two RCT subanalyses were derived from the TTM-2 trial [11] (n = 902 in the moderate/high vasopressor groups) and the HYPERION trial [12] (n = 339 with post-resuscitation shock). These studies compared TTM at 33°C with normothermia (36°C-37°C). The observational studies included a prospective registry analysis [9] (n = 145), a propensity-matched historic control study [8] (n = 40), and a single-arm interventional study [22] (n = 50). The observational studies utilized a historic or non-concurrent control group treated with standard normothermia.

The definitions of CS varied slightly across studies but included hypotension (systolic blood pressure (SBP) < 90 mmHg or mean arterial pressure < 65 mmHg) requiring vasopressor support and/or signs of end-organ hypoperfusion (e.g., elevated lactate levels and cool extremities). While definitions were study-specific and not formally harmonized, all included studies required objective evidence of hypotension (SBP < 90 mmHg) or the necessity of vasopressor support to maintain perfusion pressure. The baseline characteristics of the included studies are summarized in Table 1. Detailed quantitative metrics of shock severity, such as the Vasoactive Inotropic Score (VIS) or lactate clearance kinetics, were not uniformly reported across all observational studies, limiting direct comparison of baseline illness severity beyond the binary presence of shock.

**Table 1:** Characteristics of included studies AMI: acute myocardial infarction; CI: cardiac index; CS: cardiogenic shock; IABP: intra-aortic balloon pump; Obs: observational; OHCA: out-of-hospital cardiac arrest; PCI: percutaneous coronary intervention; RCT: randomized controlled trial; SBP: systolic blood pressure; TTM: targeted temperature management; VF: ventricular fibrillation *Sample size for the "moderate vasopressor" subgroup.

Study	Design	Population (shock definition)	Intervention	Comparator	Sample size (TTM/control)	Primary outcome
Zobel et al. [8]	Obs (matched)	OHCA with CS (SBP < 90, CI ≤ 2.2)	33°C (24 h)	Historic normothermia	20/20	Mortality (6-mo)
Orban et al. [9]	Obs (registry)	AMI + CS + PCI	32°C-34°C (24 h)	No TTM	64/81	Mortality (30-day)
Hovdenes et al. [22]	Obs (single-arm)	OHCA + VF + PCI (inc. IABP use)	32°C-34°C (24 h)	None	50 (single arm)	Survival (6-mo)
Düring et al. [11]	RCT (post hoc)	OHCA with vasopressor support	33°C (28 h)	Normothermia (≤37.8°C)	448/454*	Mortality (180-day)
Ziriat et al. [12]	RCT (post hoc)	Non-shockable + post-resuscitation shock	33°C (24 h)	Normothermia (37°C)	159/180	Functional outcome (90-day)

Quality and Risk of Bias Assessment

**Figure 2:**
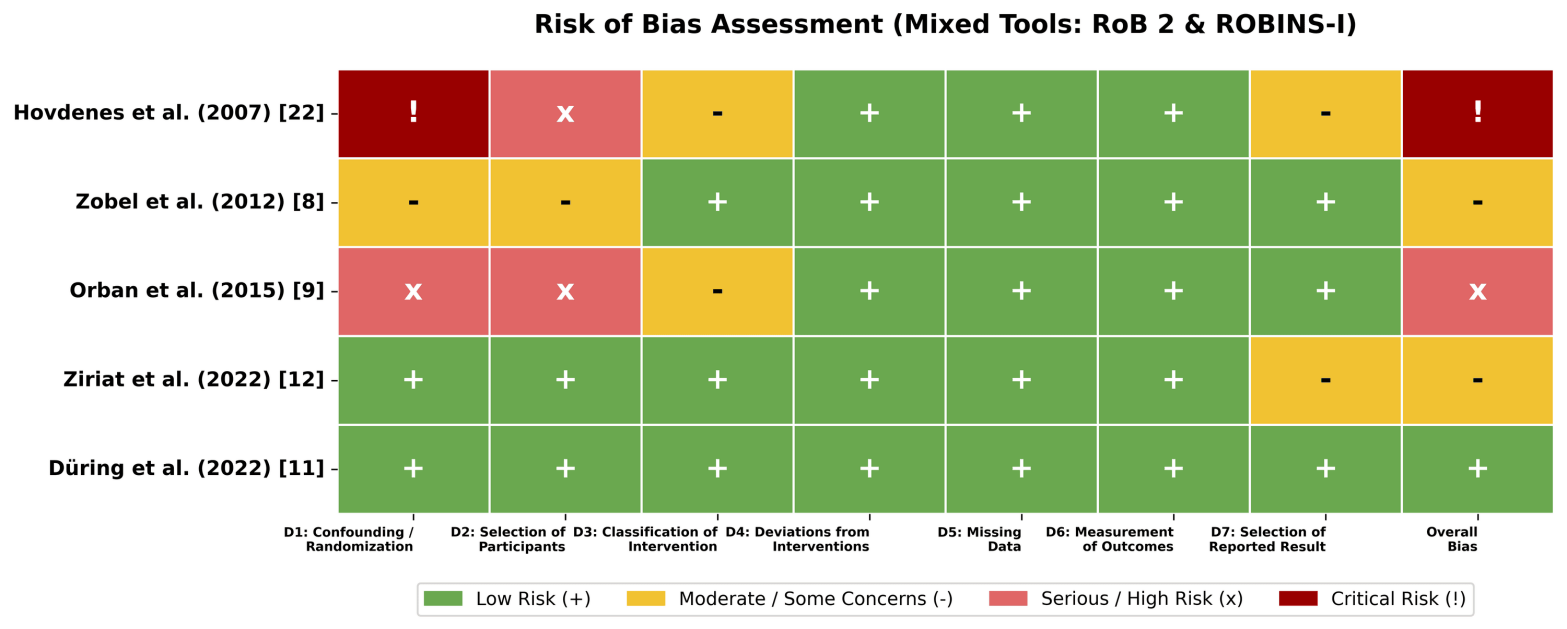
Risk of bias assessment The traffic light plot summarizes the risk of bias for each included study. RCTs were assessed using the RoB 2 tool, and observational studies were assessed using the ROBINS-I tool. Green indicates low risk, yellow indicates moderate risk or some concerns, and red indicates serious or critical risk [8,9,11,12,22]. RCT: randomized controlled trial; RoB 2: Cochrane Risk of Bias 2; ROBINS-I: risk of bias in non-randomized studies of interventions

The methodological quality of the included studies varied according to the study design. The risk of bias assessment is summarized in Figure 2. For the randomized data, post hoc analyses of the TTM-2 [11] and HYPERION [12] trials were evaluated using the RoB 2 tool. Düring et al. [11] were adjudicated as having a low risk of bias, given the robust randomization, concealed allocation, and blinded outcome assessment of the parent trial. Ziriat et al. [12] were rated as having some concerns in the domain of selection of the reported result, due to the post hoc nature of the subgroup analysis, which may introduce data-driven selection bias despite the high quality of the parent trial.

The ROBINS-I tool was used to assess the observational studies. Zobel et al. [8] were rated as having moderate risk; although propensity matching was employed to balance baseline characteristics, the small sample size (n = 40) limits the ability to fully control for unmeasured confounding. Orban et al. [9] were rated as serious risk due to confounding by indication, as the decision to implement TTM was at the physician's discretion, potentially selecting patients with different prognoses. Hovdenes et al. [22] was rated as having critical risk for comparative effectiveness, as it was a single-arm study comparing outcomes against historic controls without adjustment for temporal changes in critical care management.

Visual inspection of the risk of bias distribution (Figure 2) highlights a clear dichotomy between high-quality evidence from recent RCTs and lower-quality evidence from earlier observational cohorts. This gradient in study quality is a critical factor in interpreting the differences in results between study types.

Primary Outcome: All-Cause Mortality

**Figure 3:**
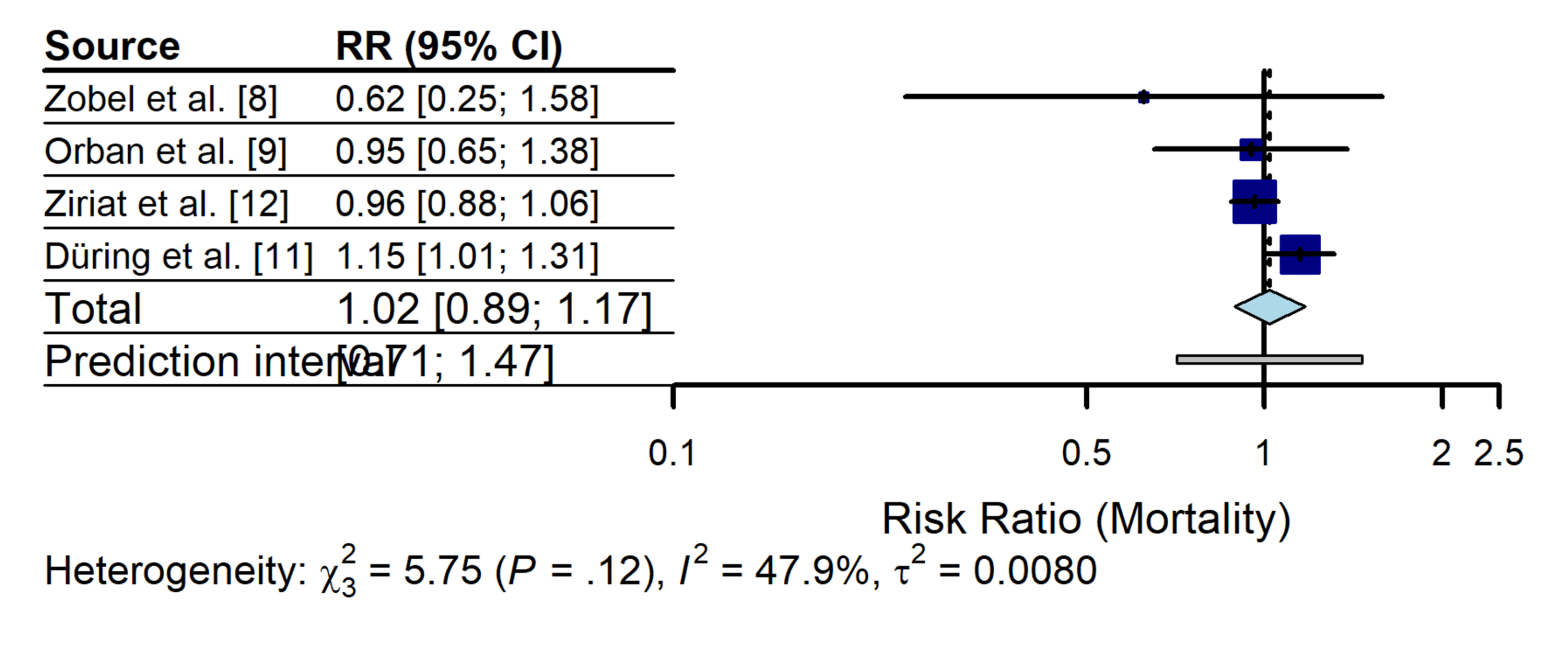
Forest plot of the primary outcome (all-cause mortality) comparing TTM versus normothermia/control in patients with cardiogenic shock The pooled estimate was calculated using a random-effects model (DerSimonian-Laird method). Squares represent the point estimate of each study with their size proportional to the weight of the study. Horizontal lines represent 95% confidence intervals (CIs). The diamond represents the pooled risk ratio (RR). TTM: targeted temperature management

Data on all-cause mortality were available for four comparative studies involving 1,446 patients (n = 675 in the TTM group and n = 771 in the control group) [8,9,11,12]. Using a random-effects model (DerSimonian-Laird), the pooled analysis revealed that mortality rates did not differ significantly between the group receiving TTM at 32°C-34°C (305/675 events (45.2%)) and the group managed with normothermia or no temperature control (347/771 events (45.0%)) (RR 1.02; 95% CI 0.89 to 1.17; p = 0.76) (Figure 3).

The point estimates varied considerably according to the study design. The small observational study by Zobel et al. [8] favored TTM (RR 0.62; 95% CI 0.25 to 1.58), whereas the large randomized trial subanalysis by Düring et al. [11] suggested a trend toward harm (RR 1.15; 95% CI 1.01 to 1.31). Ziriat et al. [12] showed a neutral effect (RR 0.96; 95% CI 0.88 to 1.06). This divergence resulted in moderate statistical heterogeneity (I^2^ = 47.9 %; χ^2^ = 5.75, p = 0.12).

**Figure 4:**
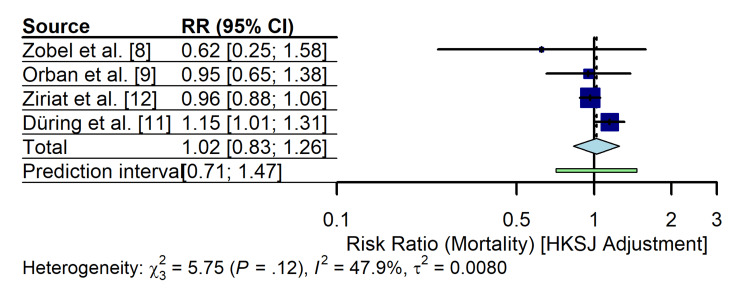
Sensitivity analysis forest plot using the HKSJ adjustment This method provides more conservative confidence intervals (CIs) to account for the small number of studies and heterogeneity. The results remain consistent with the primary analysis, showing no significant mortality benefit. HKSJ: Hartung-Knapp-Sidik-Jonkman; RR: risk ratio

To ensure the robustness of this finding, given the small number of studies and heterogeneity, a sensitivity analysis using the HKSJ adjustment was performed. The HKSJ method yielded a similar point estimate but with a wider CI (RR 1.02; 95% CI 0.83 to 1.26; p = 0.77), further confirming the lack of a statistically significant mortality benefit (Figure 4). Analysis using ORs yielded consistent results (OR 1.01; 95% CI 0.70 to 1.46).

The single-arm study by Hovdenes et al. [22] reported a mortality rate of 18% (9/50) in a highly selected population of patients with shockable rhythms undergoing immediate percutaneous coronary intervention (PCI), which is lower than the 40%-50% mortality rates observed in the broader shock populations of the comparative studies.

Secondary Outcomes

Neurological function: Neurological outcomes were synthesized narratively rather than quantitatively due to variations in outcome scales (CPC vs. mRS) and reporting formats. Neurological outcomes were reported using the CPC or mRS at variable time points ranging from hospital discharge to six months. In the RCT subanalyses, neither Düring et al. [11] nor Ziriat et al. [12] found a significant benefit of hypothermia on favorable functional outcomes (defined as CPC 1-2 or mRS 0-3) in patients with shock. Ziriat et al. reported a favorable outcome in 8.8% of TTM patients vs. 5.6% of controls (p = 0.24) [12]. Conversely, the observational study by Zobel et al. [8] reported a trend toward improved neurological recovery in survivors treated with TTM, although the small sample size precluded statistical significance of the results.

**Figure 5:**
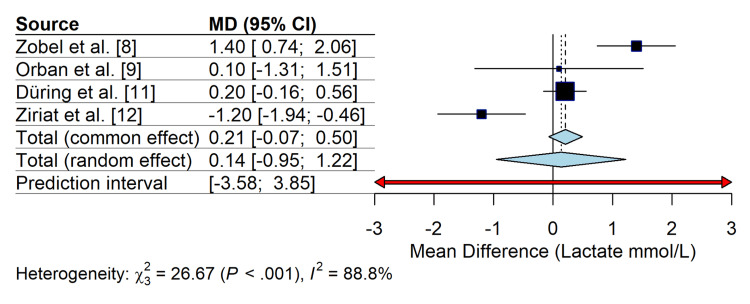
Forest plot of the mean difference (MD) in serum lactate levels (mmol/L) between TTM and control groups Positive values indicate higher lactate levels in the TTM group. The analysis shows no significant difference in lactate clearance, though substantial heterogeneity exists between studies. TTM: targeted temperature management; CI: confidence interval

Physiological parameters: serum lactate levels: Serum lactate levels, which are surrogates for tissue perfusion and shock severity, were analyzed in four studies [8,9,11,12]. Due to inconsistent reporting of clearance rates, the analysis compared absolute mean serum lactate levels measured at 12-24 hours post-admission. The pooled analysis of the MD in lactate levels (measured at 12-24 hours post-admission or peak levels) showed no significant difference between the TTM and control groups (MD 0.21 mmol/L; 95% CI -0.07 to 0.50; p = 0.14) (Figure 5). However, significant heterogeneity was observed (I^2^ = 88.8%), largely driven by Ziriat et al. [12], where the control group had higher lactate levels, suggesting a potential baseline imbalance or different shock severity.

Safety and adverse events: Adverse events specific to the shock population were reported. Düring et al. [11] noted a significantly higher incidence of arrhythmias resulting in hemodynamic compromise in the hypothermia group compared to normothermia (24% vs. 17%, p < 0.001) in the overall TTM-2 trial, a finding that persisted in the shock subgroups. Orban et al. [9] reported a trend toward higher rates of bleeding (thrombolysis in myocardial infarction (TIMI) major: 25% vs. 12%, p = 0.07) and stent thrombosis in the TTM group. Zobel et al. [8] observed a significant increase in SVR during cooling, which may increase cardiac afterload and be potentially detrimental in CS, although this was managed with vasodilator therapy.

Heterogeneity and Subgroup Analyses

**Figure 6:**
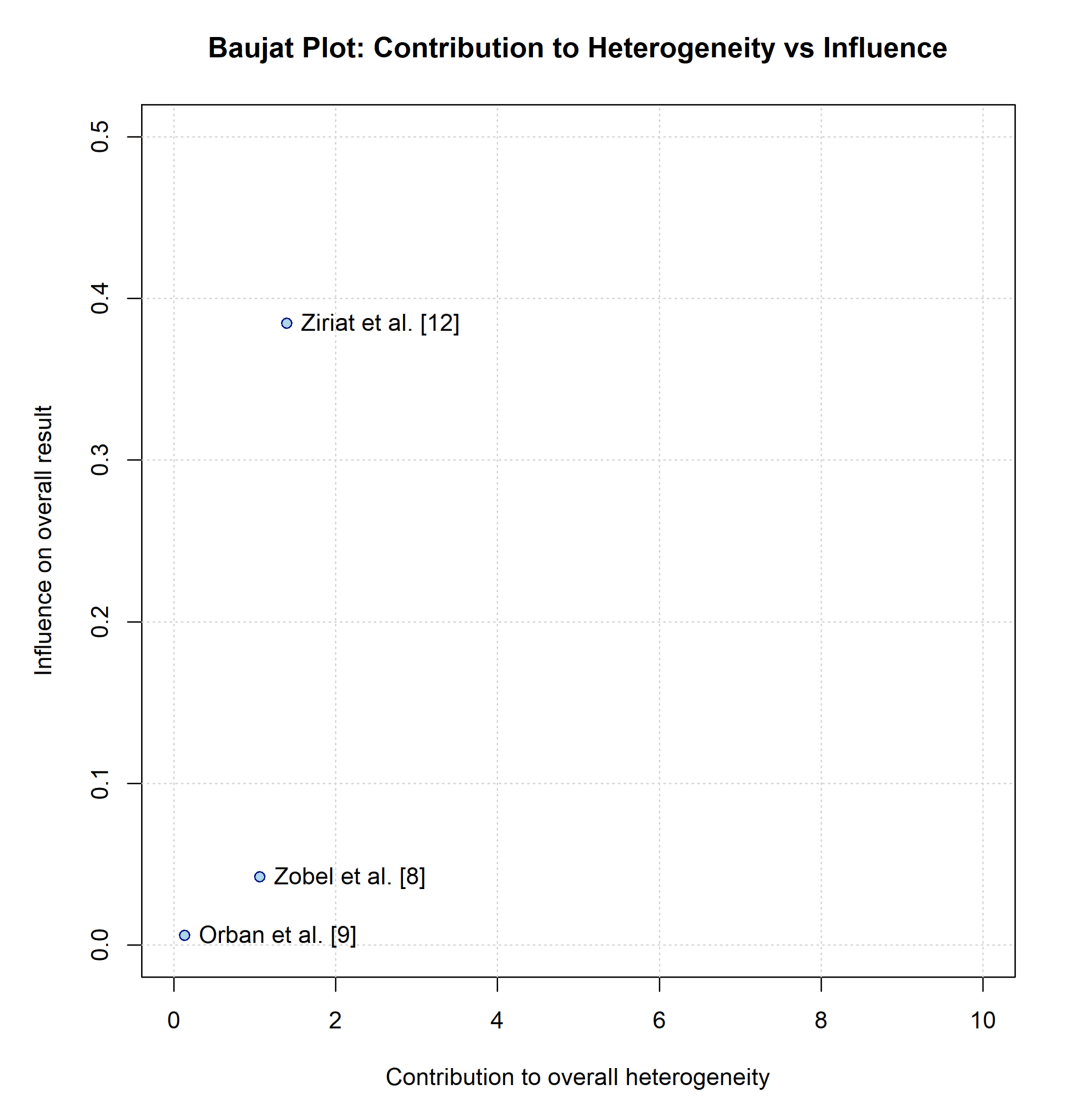
Baujat plot exploring the contribution of each study to the overall heterogeneity (X-axis) versus its influence on the pooled result (Y-axis) Studies in the upper-right quadrant (e.g., Ziriat et al. [12]) contribute most to the instability of the meta-analysis result.

Sources of heterogeneity: Moderate statistical heterogeneity regarding the primary mortality endpoint was observed (I^2^ = 47.9%; χ^2^ = 5.75, p = 0.12). A Baujat plot was constructed to explore the origins of this variance (Figure 6). The plot identified Zobel et al. [8] and Düring et al. [11] as the primary contributors to the overall heterogeneity, as Zobel et al. contributed due to their large effect size favoring TTM (RR 0.62), which diverged from the neutral-to-harmful effect observed in the much larger Düring et al. study (RR 1.15). This finding reinforces the hypothesis that the study design and sample size are key drivers of the observed inconsistency. Further exploration of heterogeneity based on arrest rhythm (shockable vs. non-shockable) or quantitative shock severity was precluded by the lack of stratified data within the primary studies.

**Figure 7:**
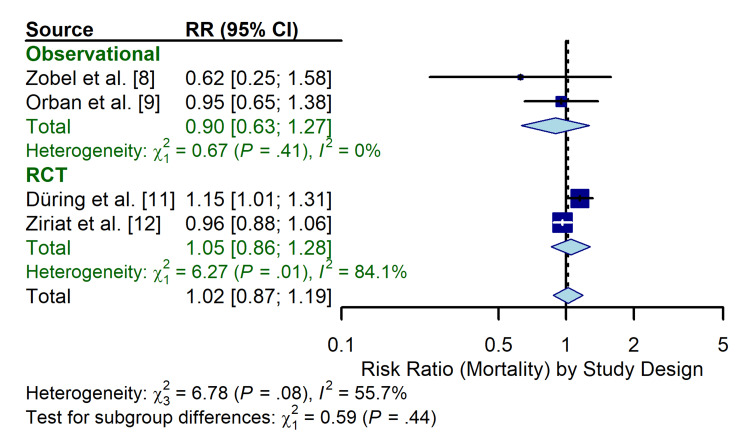
Forest plot of subgroup analysis stratified by study design (observational vs. RCT) RCT: randomized controlled trial; RR: risk ratio; CI: confidence interval

Subgroup analysis: study design: A pre-specified subgroup analysis stratified by study design (RCT vs. observational) revealed a distinct dichotomy in the treatment effect (Figure 7). The observational studies (n = 2) showed a trend toward benefit with TTM (pooled RR 0.90; 95% CI 0.63 to 1.27; I^2^ = 0%), while the RCTs (n = 2) showed a trend toward harm or neutrality (pooled RR 1.05; 95% CI 0.86 to 1.28; I^2^ = 84%). Although the test for subgroup differences was not statistically significant (χ^2^ = 0.59, p = 0.44) due to low power, the point estimates suggested that the apparent benefit of TTM may be exaggerated in non-randomized settings.

**Figure 8:**
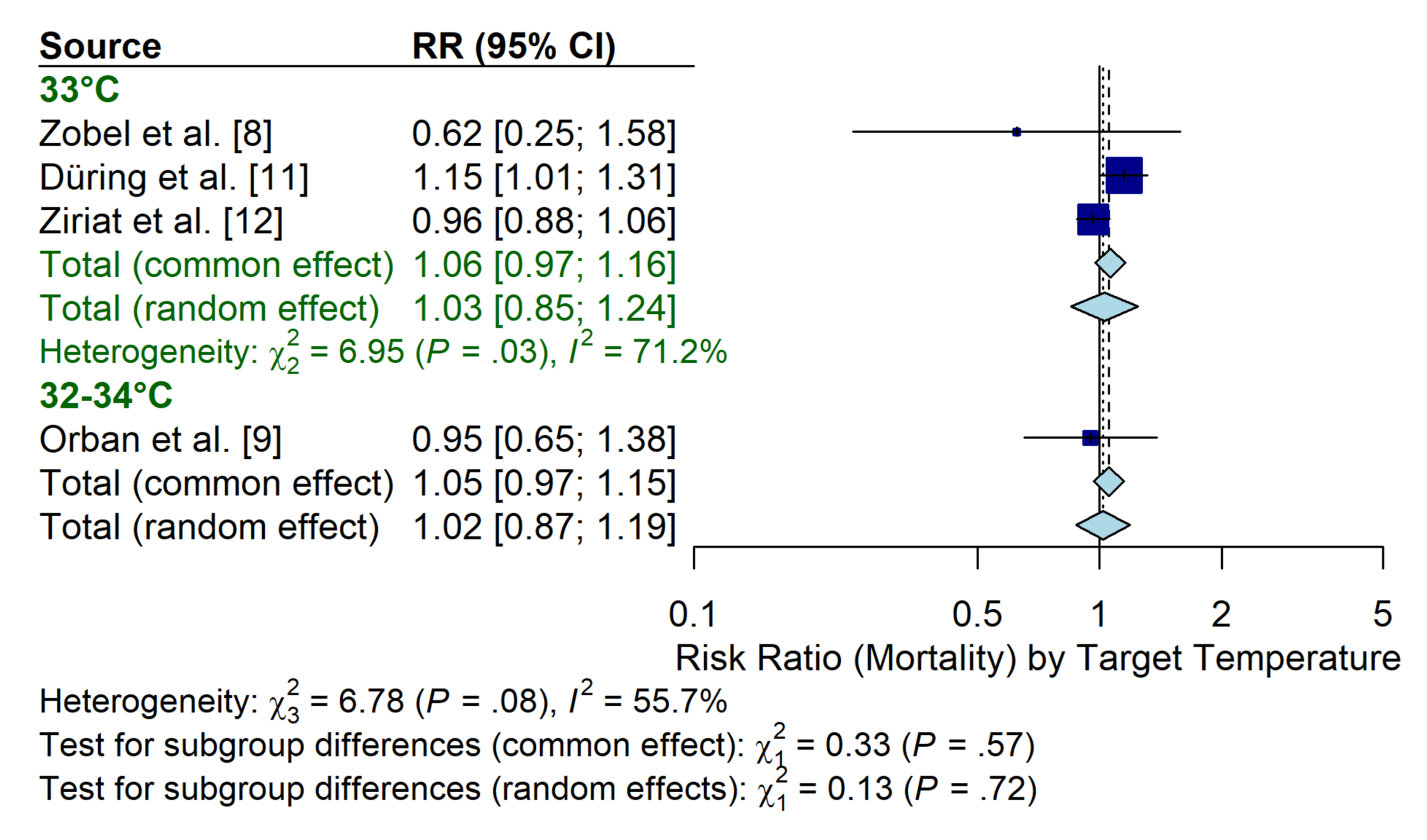
Forest plot of subgroup analysis stratified by target temperature (33°C vs. 32°C-34°C) RR: risk ratio; CI: confidence interval

Subgroup analysis: target temperature: Also, studies were stratified by the target temperature utilized: strict 33°C (used in [8,11,12]) versus a range of 32°C-34°C (used in [9]). The results were consistent across temperature targets, with no significant difference in mortality between the 33°C group (RR 1.06; 95% CI 0.97 to 1.16) and the 32°C-34°C group (RR 0.95; 95% CI 0.65 to 1.38) (test for subgroup differences: p = 0.57) (Figure 8).

**Figure 9:**
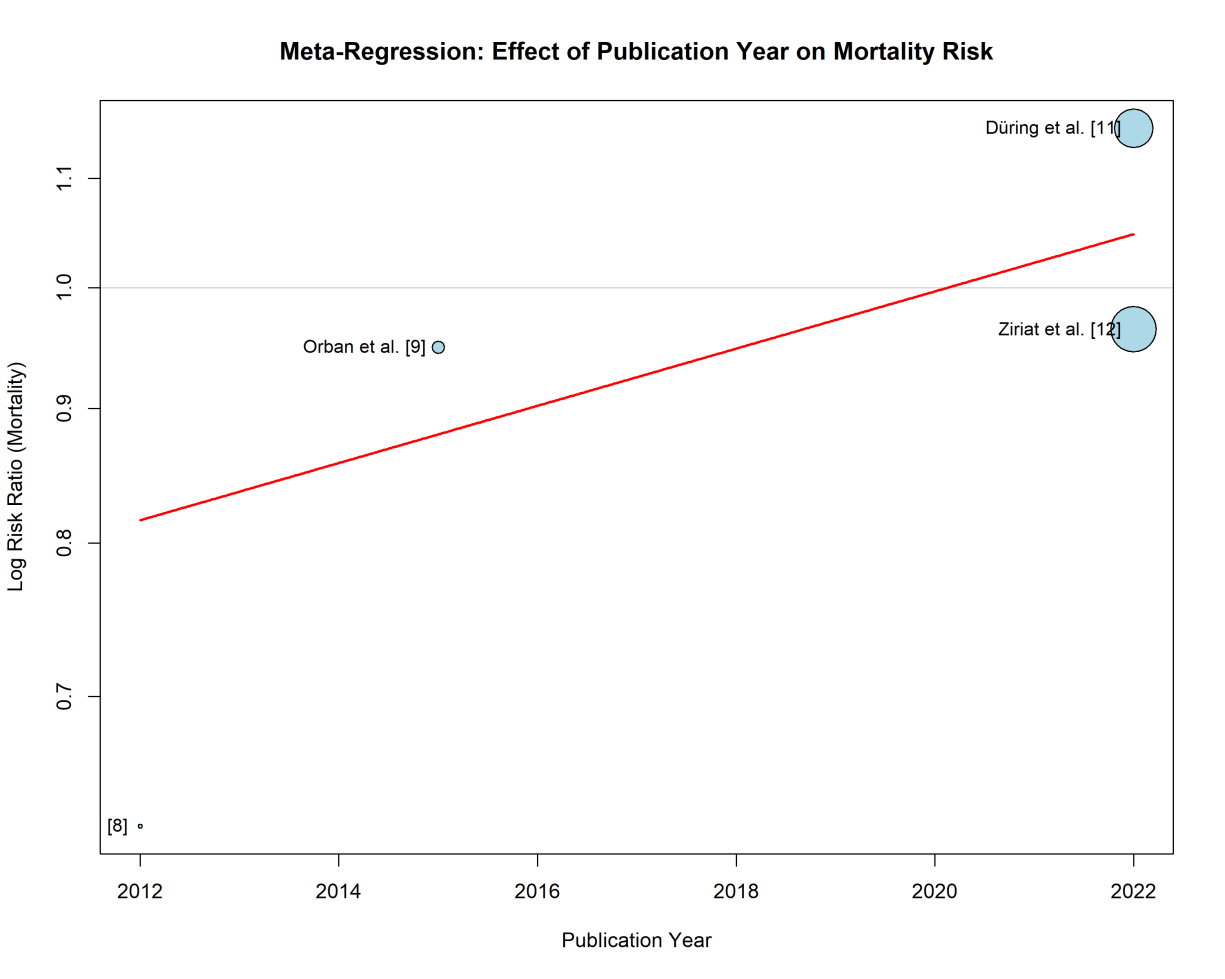
Bubble plot of meta-regression analysis showing the relationship between publication year (X-axis) and the log risk ratio for mortality (Y-axis) The size of each bubble is proportional to the weight of the study. The regression line (red) indicates a trend where more recent studies report less favorable outcomes for TTM compared to earlier studies. TTM: targeted temperature management

Meta-regression: publication year: The meta-regression analysis examining the effect of publication year on the RR for mortality demonstrated a positive slope (Figure 9). Older studies (e.g., 2012) were associated with lower RRs (favoring TTM), whereas more recent studies (2022) reported RRs greater than 1.0. This temporal trend suggests a "proteomics of time" effect, where early, smaller studies with potential selection bias reported large benefits that have diminished in the era of high-quality, large-scale RCTs.

Assessment of Reporting and Dissemination Biases

**Figure 10:**
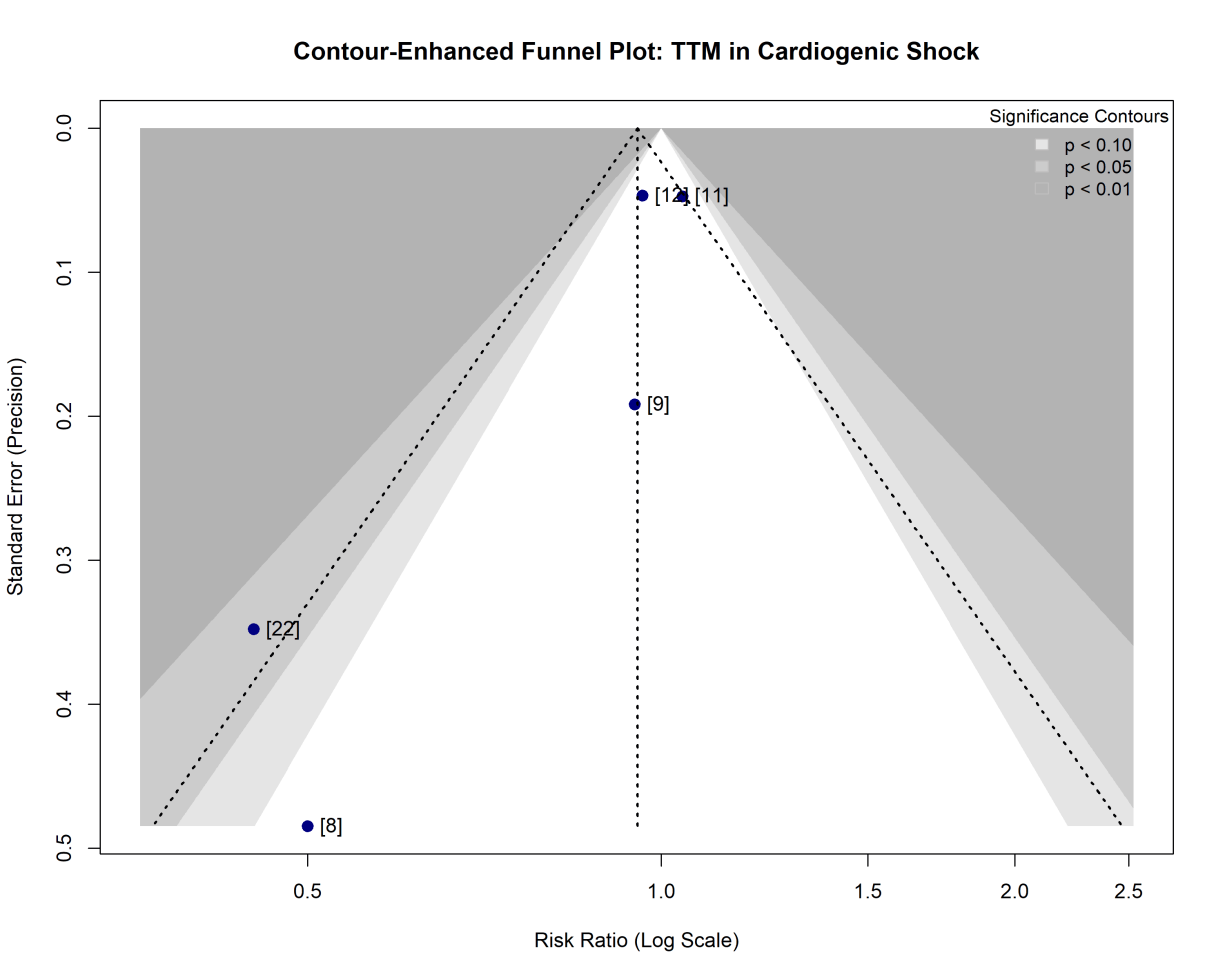
Contour-enhanced funnel plot for the assessment of publication bias The pooled treatment effect is indicated by the vertical dashed line. The shaded regions represent levels of statistical significance (p < 0.10, p < 0.05, and p < 0.01). The asymmetry, with small studies falling into the significant benefit region (left) and large studies in the non-significant region (top center), suggests potential publication bias or small-study effects. TTM: targeted temperature management

Small-study effects and publication bias: Visual inspection of the contour-enhanced funnel plot (Figure 10) revealed an asymmetry in the distribution of study effects. The smaller, lower-precision studies (e.g., Zobel et al. [8]) were located in the lower-left quadrant, falling within the region of statistical significance (p < 0.05) and indicating a strong beneficial effect of TTM. Conversely, the larger, high-precision studies (Düring et al. [11], Ziriat et al. [12]) were clustered near the top of the funnel, centered around the line of no effect (RR ≈ 1.0). Notably, small studies were absent from the bottom-right quadrant (outcomes favoring control or harm), suggesting that small studies with negative or neutral results may not have been published or identified.

Statistical tests for bias: Quantitative assessment using Harbord’s modified test for binary data yielded a p-value of 0.70, while Peters’ test yielded a p-value of 0.79. Although these tests do not formally reject the null hypothesis of symmetry, they are known to have low power when the number of studies is small (n < 10). Therefore, the visual evidence of asymmetry in the funnel plot, combined with the clear discrepancy between observational and randomized evidence seen in the subgroup analysis, strongly suggests the presence of small-study effects or selection bias in earlier literature.

Certainty of Evidence (GRADE)

The GRADE framework was applied to evaluate the certainty of evidence regarding all-cause mortality. The overall evidence quality was very low. The evidence profile was downgraded for three key reasons: two of the four studies included in the meta-analysis were observational studies with moderate to serious risk of bias due to confounding by indication and lack of randomization [8,9]. There was substantial heterogeneity (I^2^ = 47.9%) in the point estimates, with observational studies suggesting a large benefit (RR ~0.60) and RCTs suggesting neutrality or harm (RR ~1.15). The CIs of the individual studies did not completely overlap. Although the total sample size was adequate (n = 1,446), the 95% CI for the pooled RR (0.89 to 1.17) crossed the line of no effect and included both the possibility of appreciable benefit and clinically significant harm. Imprecision was identified as the 95% CI crossed the line of no effect and included both potential benefit and harm, rendering the calculation of optimal information size secondary. Indirectness was not considered a downgrading factor, as the included populations directly represented the clinical phenotype of CS despite minor variations in definitions.

When stratified by study design, the certainty of the RCT evidence alone was moderate (downgraded only for imprecision), whereas the observational evidence was very low due to risk of bias and inconsistency. However, the overall rating for the pooled body of evidence remains very low.

The summary of the findings (Table 2) details the absolute and relative effects, along with the certainty assessment for each outcome. Based on this very low certainty evidence, it cannot be concluded that TTM at 32°C-34°C reduces mortality in patients with CS compared to normothermia.

**Table 2:** GRADE summary of findings CI: confidence interval; RR: risk ratio; TTM: targeted temperature management; GRADE: Grading of Recommendations Assessment, Development, and Evaluation ^a^Downgraded one level. Two of the four included studies are observational cohorts with moderate to serious risk of bias due to lack of randomization and potential confounding by indication [8,9]. ^b^Downgraded one level. There is substantial heterogeneity (I^2^ = 47.9%) in the point estimates. Observational studies consistently favored TTM (RR range 0.62–0.95), whereas the randomized trial analyses indicated a neutral or potentially harmful effect (RR range 0.96–1.15). ^c^Downgraded one level. The 95% CI crosses the line of no effect (1.0) and includes both the possibility of appreciable benefit (11% reduction) and clinically significant harm (17% increase).

Outcome	Anticipated absolute effects (95% CI)	Relative effect (95% CI)	No. of participants (studies)	Certainty of the evidence (GRADE)
All-cause mortality (follow-up: hospital discharge to 6 months)	Risk with normothermia: 555 per 1,000 risk difference with TTM: 11 more deaths per 1,000 (from 61 fewer to 94 more)	RR 1.02 (0.89 to 1.17)	1,446 (4 studies) [8,9,11,12]	⨁◯◯◯ very low^a,b,c^

Discussion

This study represents the most extensive meta-analysis currently available regarding TTM application in cardiac arrest survivors who present with concurrent CS. The analysis of 1,446 patients from five studies found no significant association between TTM at 32°C-34°C and reduced all-cause mortality compared to normothermia or no temperature control. Furthermore, no significant differences were observed in favorable neurological outcomes or lactate clearance. However, these aggregate findings mask a critical dichotomy in the evidence base: while earlier, smaller observational studies suggested a robust survival benefit, recent high-quality RCT data have shown a neutral or potentially harmful signal. The overall certainty of the evidence remains very low, precluding a definitive recommendation for moderate hypothermia in this high-risk population.

Divergence Between Observational and Randomized Evidence

A central finding of this study is the stark contrast between the study designs. The observational data, exemplified by Zobel et al. [8] and Orban et al. [9], suggested that TTM might be life-saving in shock, with RRs ranging from 0.62 to 0.95. This effect size is biologically plausible, given the theoretical benefits of hypothermia in reducing metabolic demand and mitigating myocardial reperfusion injury [1,2]. However, these studies are limited by selection bias; patients selected for cooling were younger or had more favorable prognostic factors than those who were not, despite attempts at statistical adjustments.

In contrast, the subanalyses of the landmark TTM-2 [11] and HYPERION [12] trials, which benefited from randomization and blinded outcome assessment, failed to replicate this benefit. The TTM-2 analysis by Düring et al. suggested a signal toward harm (RR 1.15) in patients with moderate vasopressor support [11]. This "proteomics of time" effect, where therapeutic efficacy diminishes as trial rigor increases, is a well-documented phenomenon in the critical care literature. Beyond study design, this likely reflects the evolution of post-resuscitation care over the last decade. Improvements in early coronary angiography, standardized vasopressor strategies, and mechanical circulatory support (MCS) may have improved baseline survival, thereby diminishing the apparent relative benefit of hypothermia observed in older, less standardized cohorts. This meta-regression confirmed this temporal trend, showing that more recent studies reported significantly less favorable outcomes for TTM than older studies.

Physiological Trade-Offs in CS

The lack of benefit in the shock population may be explained by the competing physiological effects of hypothermia. While cerebral protection is the primary goal, hypothermia induces bradycardia, increases SVR, and can impair myocardial contractility and diastolic function [7,8]. In a patient with preserved cardiac function, these effects are manageable. However, in a patient with CS, the increase in afterload SVR combined with bradycardia may further compromise cardiac output, requiring higher doses of vasopressors and exacerbating end-organ hypoperfusion. This hypothesis is supported by the TTM-2 data, which showed a significantly higher rate of hemodynamic instability and arrhythmias in the hypothermia group [11].

Clinical Implications

Current international guidelines recommend TTM for comatose survivors of cardiac arrest but do not offer specific recommendations for the subpopulation with CS [5]. These findings suggest that the blanket application of moderate hypothermia (33°C) in patients with profound shock should be approached cautiously. The potential for hemodynamic destabilization may outweigh its theoretical neuroprotective benefits. Clinicians must weigh these risks carefully, perhaps favoring a strategy of strict normothermia (fever prevention), which preserves hemodynamic stability while avoiding the metabolic costs of fever.

Limitations

The number of eligible studies was small (n = 5), limiting the power of subgroup analyses and tests for publication bias. The definition of "cardiogenic shock" varied slightly across studies, ranging from vasopressor requirement to specific cardiac index cut-offs. In addition, we could not control the specific method of cooling (intravascular vs. surface) or the speed of rewarming, factors which may influence hemodynamic stability. The "control" groups in the observational studies were often historical, introducing temporal bias related to improvements in general critical care over the last decade. Furthermore, residual confounding related to the use of MCS and arrest rhythm could not be fully addressed due to the lack of stratified data in observational cohorts. We were unable to assess potential dose-response relationships regarding the duration of hypothermia, as most included protocols utilized a standard 24-hour cooling period.

The meta-regression analysis examining the effect of publication year is limited by the small number of studies (n = 5); these findings are exploratory and susceptible to overfitting, precluding definitive conclusions regarding temporal trends. Furthermore, the majority of patients in the included studies suffered from OHCA; therefore, these findings should be applied with caution to in-hospital cardiac arrest (IHCA) populations where the etiology and shock trajectory may differ.

## Conclusions

TTM at 32°C-34°C does not appear to improve survival or neurological outcomes in cardiac arrest survivors complicated by CS compared to normothermia. The apparent benefits seen in early observational studies have not been borne out by recent high-quality randomized evidence, which raises concerns about potential hemodynamic harm. Until dedicated RCTs specifically target this high-risk population, ideally stratifying patients by shock severity (e.g., SCAI (Society for Cardiovascular Angiography and Interventions) stages), strict normothermia may be a safer and equally effective strategy for cardiac arrest survivors with moderate-to-severe shock dependent on vasopressors.

## References

[1] Neumar RW, Nolan JP, Adrie C (2008). Post-cardiac arrest syndrome: epidemiology, pathophysiology, treatment, and prognostication. A consensus statement from the International Liaison Committee on Resuscitation (American Heart Association, Australian and New Zealand Council on Resuscitation, European Resuscitation Council, Heart and Stroke Foundation of Canada, InterAmerican Heart Foundation, Resuscitation Council of Asia, and the Resuscitation Council of Southern Africa); the American Heart Association Emergency Cardiovascular Care Committee; the Council on Cardiovascular Surgery and Anesthesia; the Council on Cardiopulmonary, Perioperative, and Critical Care; the Council on Clinical Cardiology; and the Stroke Council. Circulation.

[2] Hypothermia after Cardiac Arrest Study Group (2002). Mild therapeutic hypothermia to improve the neurologic outcome after cardiac arrest. N Engl J Med.

[3] Bernard SA, Gray TW, Buist MD, Jones BM, Silvester W, Gutteridge G, Smith K (2002). Treatment of comatose survivors of out-of-hospital cardiac arrest with induced hypothermia. N Engl J Med.

[4] Nielsen N, Wetterslev J, Cronberg T (2013). Targeted temperature management at 33°C versus 36°C after cardiac arrest. N Engl J Med.

[5] Dankiewicz J, Cronberg T, Lilja G (2021). Hypothermia versus normothermia after out-of-hospital cardiac arrest. N Engl J Med.

[6] Lascarrou JB, Merdji H, Le Gouge A (2019). Targeted temperature management for cardiac arrest with nonshockable rhythm. N Engl J Med.

[7] Magder S (2021). Cardiogenic shock part 1: epidemiology, classification, clinical presentation, physiological process, and nonmechanical treatments. Cardiopulmonary Monitoring.

[8] Zobel C, Adler C, Kranz A (2012). Mild therapeutic hypothermia in cardiogenic shock syndrome. Crit Care Med.

[9] Orban M, Mayer K, Morath T (2015). The impact of therapeutic hypothermia on on-treatment platelet reactivity and clinical outcome in cardiogenic shock patients undergoing primary PCI for acute myocardial infarction: results from the ISAR-SHOCK registry. Thromb Res.

[10] Fuernau G, Thiele H (2019). Response by Fuernau and Thiele to letters regarding article, "Mild hypothermia in cardiogenic shock complicating myocardial infarction: randomized SHOCK-COOL trial". Circulation.

[11] Düring J, Annborn M, Cariou A (2022). Influence of temperature management at 33 °C versus normothermia on survival in patients with vasopressor support after out-of-hospital cardiac arrest: a post hoc analysis of the TTM-2 trial. Crit Care.

[12] Ziriat I, Le Thuaut A, Colin G (2022). Outcomes of mild-to-moderate postresuscitation shock after non-shockable cardiac arrest and association with temperature management: a post hoc analysis of HYPERION trial data. Ann Intensive Care.

[13] Page MJ, McKenzie JE, Bossuyt PM (2021). The PRISMA 2020 statement: an updated guideline for reporting systematic reviews. BMJ.

[14] Sterne JA, Savović J, Page MJ (2019). RoB 2: a revised tool for assessing risk of bias in randomised trials. BMJ.

[15] Sterne JA, Hernán MA, Reeves BC (2016). ROBINS-I: a tool for assessing risk of bias in non-randomised studies of interventions. BMJ.

[16] DerSimonian R, Laird N (1986). Meta-analysis in clinical trials. Control Clin Trials.

[17] IntHout J, Ioannidis JP, Borm GF (2014). The Hartung-Knapp-Sidik-Jonkman method for random effects meta-analysis is straightforward and considerably outperforms the standard DerSimonian-Laird method. BMC Med Res Methodol.

[18] Higgins JP, Thompson SG, Deeks JJ, Altman DG (2003). Measuring inconsistency in meta-analyses. BMJ.

[19] Egger M, Davey Smith G, Schneider M, Minder C (1997). Bias in meta-analysis detected by a simple, graphical test. BMJ.

[20] Begg CB, Mazumdar M (1994). Operating characteristics of a rank correlation test for publication bias. Biometrics.

[21] Guyatt GH, Oxman AD, Vist GE, Kunz R, Falck-Ytter Y, Alonso-Coello P, Schünemann HJ (2008). GRADE: an emerging consensus on rating quality of evidence and strength of recommendations. BMJ.

[22] Hovdenes J, Laake JH, Aaberge L, Haugaa H, Bugge JF (2007). Therapeutic hypothermia after out-of-hospital cardiac arrest: experiences with patients treated with percutaneous coronary intervention and cardiogenic shock. Acta Anaesthesiol Scand.

